# The M. Powell Lawton Award Lecture: The Person-Environment Fit Framework, Older Adults, and Technology Interactions

**DOI:** 10.1093/geroni/igab046.2126

**Published:** 2021-12-17

**Authors:** Debra Dobbs

## Abstract

The lecture will be given by the 2020 recipient, Sara Czaja, PhD, FGSA of Weill Cornell Medicine. The 2021 M. Powell Lawton Award recipient is David Roth, Phd, FGSA, of Johns Hopkins University. The M. Powell Lawton Award is presented annually to an individual who has made outstanding contributions from applied research that has benefited older people and their care. The Lawton Award is generously funded by the Polisher Research Institute of Abramson Senior Care.

